# (+)-Usnic acid and its salts, inhibitors of SARS‐CoV‐2, identified by using in silico methods and in vitro assay

**DOI:** 10.1038/s41598-022-17506-3

**Published:** 2022-07-30

**Authors:** Eunseok Oh, Weihong Wang, Kyu-Hyung Park, Chanyoon Park, Youbin Cho, JunI Lee, Eunmo Kang, Heonjoong Kang

**Affiliations:** 1 Laboratory of Marine Drugs, School of Earth and Environmental Sciences, Seoul National University, NS-80, Seoul, 08826 Korea; 2 Research Institute of Oceanography, Seoul National University, NS-80, Seoul, 08826 Korea; 3 Bio-Max Institute, Seoul National University, Seoul, 08826 Korea; 4 Interdisciplinary Graduate Program in Genetic Engineering, Seoul National University, NS-80, Seoul, 08826 Korea

**Keywords:** Infectious diseases, Computational chemistry, Drug discovery, Virtual screening

## Abstract

The pandemic caused by severe acute respiratory Coronavirus-2 (SARS-CoV-2) has been ongoing for over two years, and treatment for COVID-19, other than monoclonal antibodies, is urgently required. Accordingly, we have investigated the inhibitors of SARS-CoV-2 protein targets by high-throughput virtual screening using a marine natural products database. Considering the calculated molecular properties and availability of the compounds, (+)-usnic acid was selected as a suitable hit. In the in vitro antiviral assay of (+)-usnic acid by the immunofluorescence method, IC_50_ was 7.99 μM, which is similar to that of remdesivir used as a positive control. The generalized Born and surface area continuum solvation (MM/GBSA) method was performed to find the potent target of (+)-usnic acid, and the Mpro protein showed the most prominent value, −52.05 kcal/mol, among other SARS-CoV-2 protein targets. Thereafter, RMSD and protein–ligand interactions were profiled using molecular dynamics (MD) simulations. Sodium usnate (NaU) improved in vitro assay results with an IC_50_ of 5.33 μM and a selectivity index (SI) of 9.38. Additionally, when (+)-usnic acid was assayed against SARS-CoV-2 variants, it showed enhanced efficacy toward beta variants with an IC_50_ of 2.92 μM and SI of 11.1. We report the in vitro anti-SARS-CoV-2 efficacy of (+)-usnic acid in this study and propose that it has the potential to be developed as a COVID-19 treatment if its in vivo efficacy has been confirmed.

## Introduction

The outbreak of the pandemic coronavirus, SARS-CoV-2, in 2019 has enormously changed our way of living. SARS-CoV-2 causes COVID-19, which is a respiratory disease^1^. Because of the rapid spread of the virus and the lack of effective medication, SARS-CoV-2 has infected 500 million people worldwide with a mortality of approximately 6 million^2^. It is the third most pathogenic human coronavirus, which causes severe infection^3,4^.

SARS-CoV-2 is a single-stranded RNA virus with a genome comprising 14 open reading frames (ORFs) that encode four structural proteins, 16 nonstructural proteins (NSPs), and nine accessory proteins^5^. The structural proteins include spike (S), membrane (M), envelope (E) and nucleocapsid (N). The S glycoprotein is the main factor for SARS-CoV-2 entry into host cells. The binding of the S protein to the human ACE2 receptor cleaves the S into S1 and S2 subunits using proteases, and afterward, they are internalized through either clathrin-mediated endocytosis or plasma membrane fusion^6–8^. As the virus enters the host, the viral genome ORF1a/b is translated to pp1a and pp1ab polyproteins^9^. 16 NSPs are released by the cleavage of the proteases using papain-like protease (PLpro) and 3C-like protease (3CLpro), eventually forming the replication transcription complex (RTC)^10,11^. RTC with NSPs undergoes viral genomic RNA replication, and the transcription of subgenomic RNA produces structural proteins^10,12^. Finally, the virion is assembled through the endoplasmic reticulum (ER), ER-Golgi intermediate compartment (ERGIC), and Golgi apparatus, and thereafter, the virion is released by exocytosis^10,13,14^. Meanwhile, the point mutations in the receptor binding domain (RBD) of the S in SARS-CoV-2 yielded several deadly variants with increased susceptibility and transmissibility^15–17^.

From the perspective of the mechanism of the SARS-CoV-2 infection, the target proteins for drug discovery can be mainly categorized into two classes. The first class is the viral protein NSPs, including NSP3, NSP5, NSP12 and NSP13. The second class of target proteins is involved in the influx of viruses into the cells, originally presents in human body^18^.

NSPs play an essential role in viral replication and transcription. Of the 16 NSPs, the above-mentioned four NSPs are known as major target proteins. NSP3, also called papain-like protease (PLpro), cleaves NSP1 and NSP2 from the polyproteins pp1a and pp1ab. GRL0617, a SARS-CoV inhibitor, has been reported to exhibit in vitro antiviral efficacy against SARS-CoV-2 with IC_50_ of 2.1 μM by binding to PLpro^19,20^. NSP5, also called 3CLpro or Mpro, is the main protease involved in the maturation of other NSPs and the cleavage of polyproteins pp1a and pp1ab. MI-09 and MI-30, synthetic molecules with a five-membered bicycloproline moiety and γ-lactam ring, have shown potent inhibitory activities against SARS-CoV-2 Mpro with an IC_50_ of 15.2 and 17.2 nM respectively. Further, these compounds have shown in vivo antiviral efficacy with good PK properties and no significant toxicity^21^. NSP12, also called RNA-dependent RNA polymerase (RdRP), catalyzes the replication and transcription of the SARS-CoV-2 RNA genome. Remdesivir is an FDA-approved drug only for emergency use in COVID-19 treatment. The incorporation of remdesivir into the growing RNA product induces RdRp stalling and creates a barrier to RNA translocation^22^. NSP13 is an RNA helicase that interacts with NSP12 to enhance viral replication efficiency^23^. Posaconazole and grazoprevir have recently been identified as potential repurposing drugs that target helicase by in silico studies^24^.

The influx of the SARS-CoV-2 can be prevented by inhibiting the host proteins involved in the viral infection. Furin is the protease responsible for the proteolytic activation of the spike at the S1/S2 site^25^. Although many studies point out that furin is not the only protease involved in the cleavage of the S1/S2 site, treatment with a furin inhibitor, decanoyl-RVKR-chloromethylketone (CMK), blocks the entry of the virus^26^. TMPRSS2, a crucial protease in SARS-CoV-2 entry into the host cell, is inhibited by camostat mesilate and nafamostat, both of which are approved treatments for other medical uses. The in vitro antiviral IC_50_ of camostat mesilate is measured as 4.2 nM^27^. Further, camostat mesilate has shown in vivo antiviral activity against SARS-CoV with a survival level of above 60% when dosed in mice at 30 mg/kg^28^. Furthermore, camostat mesilate has undergone clinical trials for the treatment of COVID-19; however, it did not show any particular efficacy^29^. Cathepsin L, a lysosomal protease and a marker for various cancers, plays a crucial role in SARS-CoV-2 infection by cleaving the S protein at a position different from the furin cleavage site^30,31^. Aloxistatin (E-64d), a broad cysteine protease inhibitor, reduces the cell entry of SARS-CoV-2 pseudovirions by 92.5% at 30 μM^32^. AAK1, the numb-associated kinase (NAK) family, is responsible for clathrin-mediated endocytosis. Barcitinib is a selective Janus kinase 1/2 inhibitor that shows a high affinity for AAK1. The phase 3 clinical trial for the treatment of COVID-19 has been conducted using barcitinib and the 28-day mortality rate has been reduced by 38.2% compared to that of the placebo^33^.

Natural products have long been used to treat various diseases including cancer, metabolic diseases and infectious diseases. Natural products that hang down through folk remedies are profiled along with advances in chemistry and are regarded as useful resources for drug development^34,35^. Over 300,000 natural products obtained from various resources including plants, animals, microorganisms and marine organisms have been reported and new compounds with distinct chemical scaffolds are continually being reported^34,36^. For marine natural products that have not been thoroughly studied compared to terrestrial compounds, more than 1,000 compounds with novel skeletons have been reported every year^37^. Natural products are structurally diverse and many of them exhibit characteristic bioactivities. Among the natural products, there are many substances with antiviral effects^35^. Spirooliganone, uncinoside, baicalein and curcumin are natural products with antiviral effects^38^. Specifically, Tamiflu, a treatment for influenza A and B, is based on a natural product derived from the Chinese spice, star anise (*Illicium verum*)^39^. Thus, the use of natural products as a starting point for new drug discovery will be a viable strategy for the successful development of therapeutics that can effectively control COVID-19.

In this study, the hit compound, (+)-usnic acid, was identified by the in silico molecular docking of more than 20,000 marine natural products against eight SARS-CoV-2 targets. The antiviral efficacy of the hit compound was evaluated using immunofluorescence. To rationalize the antiviral efficacy of the hit compound, MM-GBSA and molecular dynamics (MD) approach were employed. Finally, the potency of (+)-usnic acid was examined under various conditions including the salt formation of (+)-usnic acid (sodium and potassium salts) and in vitro assays against some SARS-CoV-2 variants. Through this study, we have demonstrated that (+)-usnic acid could be a good drug lead for the treatment of the deadly COVID-19.

## Methods

### Hit discovery through high throughput virtual screening (HTVS)

An in-house database containing natural products derived from marine sources (animals, plants, bacteria, and fungi) was selected as the HTVS ligand for the SARS-CoV-2 protein targets. The in-house database contained 24,669 marine natural products. Ligands were prepared using the LigPrep software offered by Schrödinger^40^. The OPLS3e force field was applied to the ligands generating tautomers and possible states at a target pH of 7.0 ± 2.0.

The grids were generated based on the eight SARS-CoV-2 targets listed in Table 1. For the protein grid generation, the grid box was located within the centroid of the selected binding site residues. The size of the grid box was set to dock the ligands within 20 Å. The ligands were docked using the Glide module from Schrödinger with HTVS precision^41^. The molecular properties such as absorption, distribution, metabolism and excretion (ADME) of the ligands were calculated using QikProp software^42^.

**Table 1:** The SARS-CoV-2 target protein PDBs and the binding site amino acid used in grid generation.

No	Target protein	PDB ID	Binding site amino acids	Ref
1	PLpro	6WX4	Met208, Pro247, Tyr264, Tyr268	^43^
2	Mpro	7AKU	His41, Gly143, Gln189	^44,45^
3	RdRp	7BV2	Asp618, Asp760, Asp761	^22,46^
4	Helicase	7NNG	Gln404, Arg443, Arg567	^23^
5	Furin	6EQW	Leu227, Glu257	^47^
6	TMPRSS2	7MEQ	His296, Ser441, Ser460	^48^
7	CathepinL	1MHW	Gln21, Gly23, Asp162	^49^
8	AAK1	4WSQ	Asp127, Glu180, Asn181	^50^

### Compound isolation and structure confirmation

The (+)-usnic acid was isolated from the extracts of marine fungus, *Mycosphaerella* sp., which was collected from a marine sediment at Donghae-si, Korea. The fungus was cultivated in 6 L of potato dextrose broth (PDB) dissolved in seawater in 27 °C, at 140 rpm shaking incubator for 7 days. The broth was extracted with ethyl acetate and yielded 4.01 g extract. The crude extract was fractionated into 3 fractions with a silica gel column chromatography using CH_2_Cl_2_ and MeOH as solvent. Fractions were further purified by C18 HPLC (Phenomenex luna C18 column, 250 mm × 10 mm, 5 μm) using 65% CH_3_CN in H_2_O to yield 6.8 mg (+)-usnic acid. NMR spectra were measured using a Bruker Ascend 700 MHz spectrometer. Electrospray ionization source (ESI) mass data was obtained using Agilent Technologies 6120 quadrupole mass spectrometer coupled with Agilent Technologies 1260 series HPLC. The optical rotation of (+)-usnic acid was measured in chloroform using a 10 mm path length cell on a Digital Polarimeter P-2000, Jasco Inc.

### In vitro SARS-CoV-2 inhibition evaluation by immunofluorescence method

The efficacy of antiviral activity against SARS-CoV-2 was verified by an immunofluorescence method. The SARS-CoV-2 virus was obtained from the Korea Centers for Disease Control and Prevention (KCDC, βCoV/Korea/KCDC/2020) and vero cells were obtained from the ATCC (ATCC-CCL81). The specific primary antibody for anti-SARS-CoV-2 nucleocapsid (N) protein was purchased from Sino Biological and the secondary antibodies, Alexa Fluor 488 goat anti-rabbit IgG and Hoechst 3342, were purchased from Molecular Probes. All the experiments using SARS-CoV-2 were carried out in biosafety level 3 (BSL-3) containment at Institut Pasteur Korea with strict accordance of the standard protocol for Korea National Institute of Health (KNIH) and Seoul National University (SNU).

For the cell preparation, the 384-tissue culture plates were seeded with 1.2 × 10^4^ vero cells per well. After 24 h, compounds were prepared at 10 points by twofold serial dilutions in dimethyl sulfoxide (DMSO) to 50 μM and treated to the cells. One hour after the compound treatment, cells were transferred to BSL-3 facility and the viruses were infected at MOI of 0.008. The plates were incubated at 37 ℃ for 24 h. After fixing the cells with 4% paraformaldehyde (PFA), permeabilization was performed. Anti-SARS-CoV-2 N primary antibody was treated and afterward, cells were stained with Alexa Fluor 488-conjugated goat anti-rabbit IgG secondary antibody and Hoechst 33342. The fluorescent images of the infected cells were obtained using a large capacity image analysis instrument, Operetta (Perkin Elmer).

### Computational calculation of MM-GBSA and MD simulations

The extra precision (XP) docking of (+)-usnic acid was performed against eight selected SARS-CoV-2 receptors. Based on the docking studies, the Prime MM-GBSA offered by Schrödinger was used to calculate the binding-free energy of the docked ligand for each protein target^51^. The binding free energy was calculated using the following formula:

$$\Delta G\left(\mathrm{bind}\right)= \Delta G\left(\mathrm{Solv}\right) + \Delta E\left(\mathrm{MM}\right)+ \Delta G\left(\mathrm{SA}\right)$$

∆*G*(Solv) is the difference in the GBSA solvation energy of the (+)-usnic acid-protein complex compared to the sum of the energies of the uncomplexed protein and ligand. ∆*E*(MM) is the difference in the minimized energy of the (+)-usnic acid-protein complex compared to the sum of the energies of the uncomplexed protein and ligand. ∆*G*(SA) is the difference in the surface area energy compared to the sum of the energies of the uncomplexed protein and ligand. Prime MM-GBSA was operated in the OPLS3e force field using the variable dielectric surface generalized Born (VSGB) solvation model.

The thermodynamic behavior, stability and interaction of (+)-usnic acid with Mpro were assessed by MD simulations. The MD calculations were performed using Desmond software^52,53^. The MD system was built using the TIP3P 3-site water model. The system was neutralized by adding 3 sodium ions and the salt concentration was adjusted to 0.15 M using the sodium and chloride ions. For the MD simulations, an isothermal-isobaric (NPT) ensemble class was applied with the temperature and pressure set to 300 K and 1.01325 bar respectively. The Nose–Hoover chain method was employed for the thermostat method and the Martyna–Tobias–Klein method was applied to the barostat method. The total simulation time was 100 ns, at the recording interval of 100 ps. The analysis of the MD simulation was executed using the simulation interaction diagram enclosed in the Desmond module.

### Anti-SARS-CoV-2 assay of the (+)-usnic acid salts

The anti-SARS-CoV-2 assay of the salt form of (+)-usnic acid with the counter-ions, Na^+^ or K^+^, was evaluated. The salt form of (+)-usnic acid was synthesized by titration with sodium hydroxide and potassium hydroxide and, thereafter, it was lyophilized. The structure was confirmed by NMR. The efficacy of the (+)-usnic acid salts was evaluated using the immunofluorescence as described above.

### In vitro SARS-CoV-2 variants inhibition evaluation

For the in-vitro evaluation of the (+)-usnic acid against the SARS-CoV-2 variants, three major variants of concern were selected for the assessment. The three variants included alpha (UK, B.1.1.7) variant, beta (South Africa, B.1.351) and delta (India, B.1.617.2) variants. All three strains were obtained from the Korea Disease Control and Prevention Agency (KDCA). The serial codes of each variant were hCoV-19/Korea/KDCA51463/2021, hCoV-19/Korea/KDCA55905/2021 and hCoV19/Korea/KDCA119861/2021 respectively. The protocol for in vitro evaluation was identical to that of the immunofluorescence method described above.

## Results

### Analysis of the HTVS of marine natural products against SARS-CoV-2 proteins

The HTVS for marine natural products was performed using the Glide software. An in-house database containing 24,669 marine natural product structures was analyzed. The docking scores of eight SARS-CoV-2 target proteins (PLpro, Mpro, RdRp, helicase, furin, TMPRSS2, cathepin L, and AAK1) were profiled. The number of compounds that were identified as possible hits was 2,251, with a docking score below –7. Further, the compounds were filtered with ADME properties calculated by Qikprop, following the criteria suggested by Schrödinger. Twenty-seven compounds were passed as potential hits. Considering the availability and ease of supply, four natural products were analyzed at a single dose (10 μM), and (+)-usnic acid showed efficacy against SARS-CoV-2 in an in vitro antiviral assay (Fig. S3). (+)-Usnic acid was selected as the hit compound for the further analysis. The calculated properties of the (+)-usnic acid are shown in Table 2.

**Table 2:** The calculated molecular property of the selected compound, (+)-usnic acid. The molecular property includes docking scores of protein targets and predicted ADME properties (dipole, PSA, volume, SASA, QplogS, %HumanOralAbsorption).

Compound	Docking score (Glide)														
(+)**-Usnic acid**	PLpro		Mpro		RdRp		Helicase		Furin		TMPRSS2		CathepsinL		AAK1
	−6.00		−7.70		−6.24		−4.72		−4.77		−5.53		−5.40		−9.34
	**Predicted ADME properties (QikProp)**														
	MW	Dipole		PSA		volume		SASA		QPlogS		%Human OralAbsorption		Rule of Five	
	344.3	4.9		144.6		1000.3		553.1		−2.5		66.1		0	

The (+)-usnic acid used in the experiment was structurally confirmed by NMR (Fig. S7). The purity was monitored by LC–MS and confirmed that it was over 99% (Fig. S9). The optical rotation was 500.4 ([α]^25^_D_) and it was considered optically pure by comparing with standard compound.

### Evaluation of the in vitro antiviral activities of (+)-usnic acid against SARS-CoV-2

**Figure 1:**
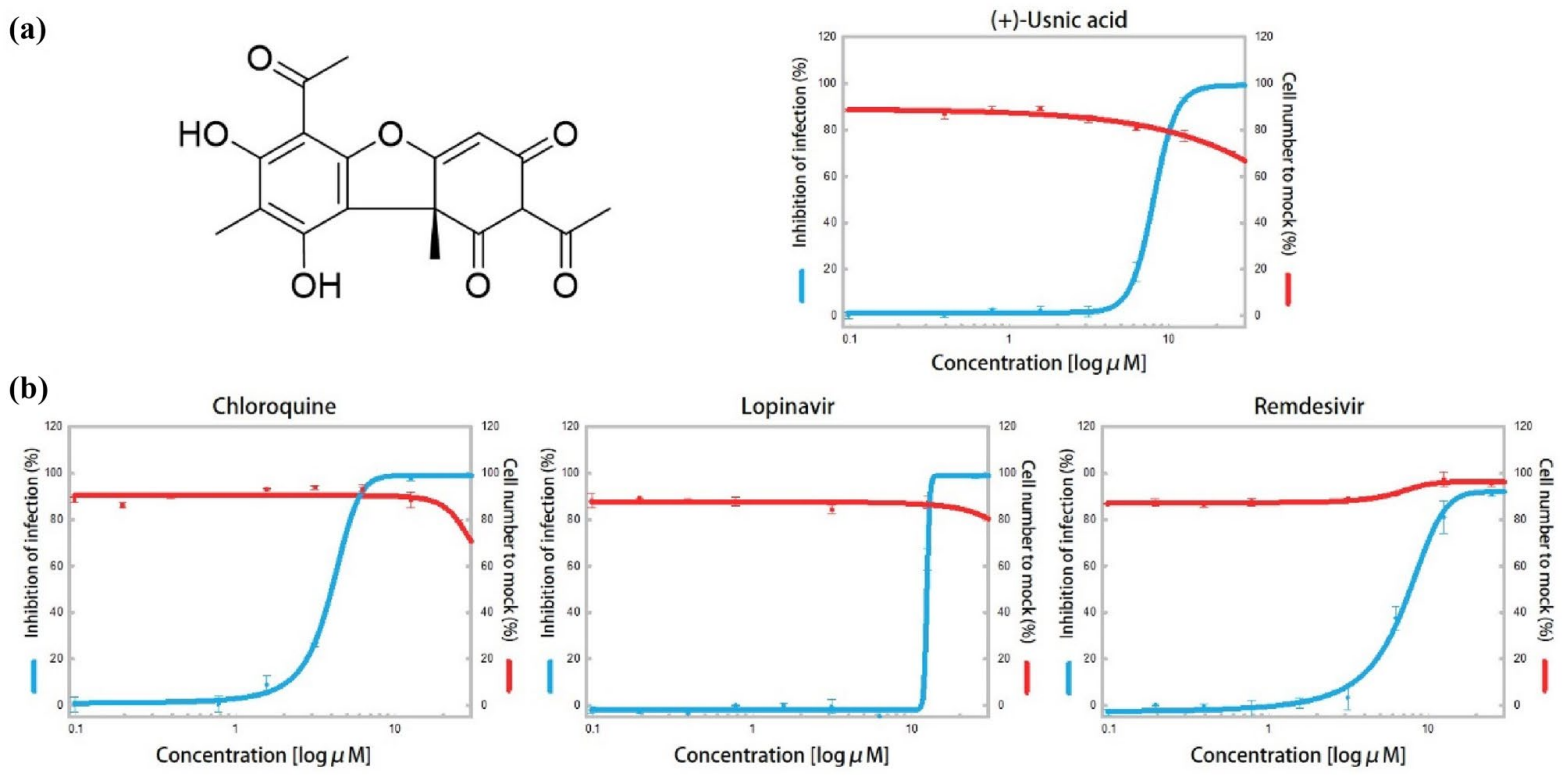
The anti-SARS-CoV-2 efficacy of (+)-usnic acid and controls; (**a**) The structure of (+)-usnic acid on the left and dose response curve of (+)-usnic acid against SARS-CoV-2 on the right; (**b**) The dose response curve of controls (chloroquine, lopinavir and remdesivir); the inhibition of infection and cell number to mock is shown using blue and red lines, respectively.

The SARS-CoV-2 antiviral efficacy of the selected compound, (+)-usnic acid, was evaluated by immunofluorescence. The dose response curves of (+)-usnic acid with three control compounds (chloroquine, lopinavir, and remdesivir) are shown in Fig. 1. The IC_50_ value of (+)-usnic acid was 7.99 μM and CC_50_ was over 50 μM. The selectivity index (SI) is calculated to be 6.26. The IC_50_ values of the controls, chloroquine, lopinavir and remdesivir, were 11.48, 11.80, and 7.42 μM, respectively. The CC_50_ values of the controls were all above 50 μM; the CC_50_ of lopinavir was higher than 150 μM. The SI of chloroquine, lopinavir and remdesivir was 13.07, 4.24, and 6.74 respectively. The efficacy of (+)-usnic acid against SARS-CoV-2 is similar to that of remdesivir, which is approved by the FDA for the treatment of emergency patients with COVID-19. The (+)-usnic acid showed a lower IC_50_ value compared to those of the two other controls, chloroquine and lopinavir.

### MM-GBSA and MD analysis of (+)-usnic acid against Mpro

**Figure 2:**
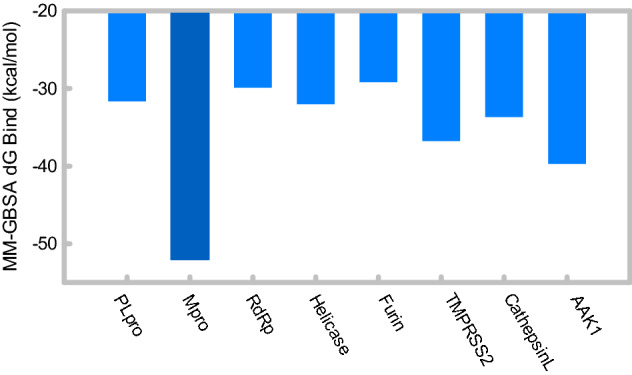
Predicted binding-free energies (kcal/mol) of 8 SARS-CoV-2 target proteins bound with (+)-usnic acid, calculated by Prime MM-GBSA.

The MM-GBSA method was used to identify the most preferable SARS-CoV-2 target proteins for (+)-usnic acid. The binding free energies are shown in Fig. 2. The target protein that showed the highest difference in the binding-free energy is Mpro, –52.05 kcal/mol. The binding free energies of remaining proteins were in the range of –39 and –29 kcal/mol, which had at least 10 kcal/mol difference with that of Mpro.

The detailed contributions of the binding-free energies are shown in Table 3. The binding free energy (dG_bind_) was calculated from the sum of dG_coloumb_, dG_covalent_, dG_Hbond_, dG_Lipo_, dG_Packing_, dG_SolvGB_ and dG_vdW_. The main contributor to Mpro, the protein with the lowest binding-free energy when complexed with (+)-usnic acid, was dG_vdW_ with a value of –45.30 kcal/mol. Further, Mpro had relatively low dG_Lipo_ compared to those of other proteins; –7.81 kcal/mol.

**Table 3:** Detailed values of the binding-free energies (kcal/mol) and binding energy states of the 8 SARS-CoV-2 target proteins that bound with (+)-usnic acid.

Protein	dG_Bind_	dG_Coulomb_	dG_Covalent_	dG_Hbond_	dG_Lipo_	dG_Packing_	dG_SolvGB_	dG_vdW_
PLpro	−31.66	−15.02	4.41	−1.05	−3.57	0.00	12.68	−29.11
Mpro	−52.05	−19.16	1.43	−1.93	−7.81	−2.35	23.07	−45.30
RdRp	−29.88	−11.91	2.38	−0.69	−3.80	−3.52	24.06	−36.41
Helicase	−32.00	−26.05	6.96	−2.66	−3.23	−3.18	27.27	−31.10
Furin	−29.16	−12.82	1.18	−1.27	−5.57	−1.08	18.86	−28.46
TMPRSS2	−36.75	−15.91	−0.34	−1.13	−5.46	0.00	18.08	−31.99
CathepsinL	−33.66	−12.27	0.34	−0.94	−6.36	−2.44	20.62	−32.62
AAK1	−39.70	−15.43	2.05	−1.31	−8.17	0.00	21.70	−38.54

**Figure 3:**
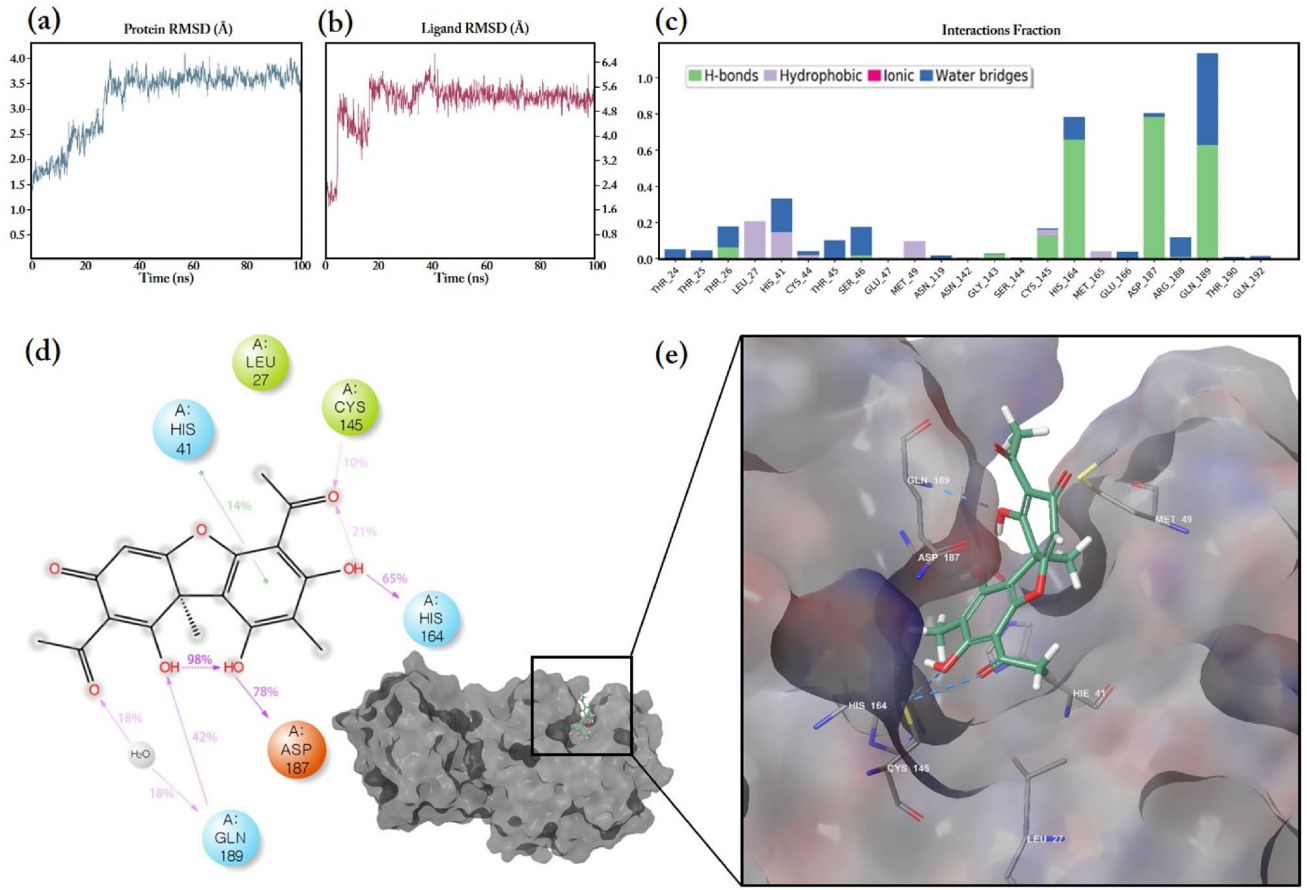
The MD simulation of (+)-usnic acid bound to the SARS-CoV-2 Mpro protein; (**a**) RMSD of the alpha-carbon of Mpro; (**b**) The ligand RMSD of (+)-usnic acid bound to Mpro, simulated for 100 ns; (**c**) The Mpro amino acid interaction with (+)-usnic acid showing H-bonds, hydrophobic interactions and water bridges; (**d**) The 2D % interaction illustration of (+)-usnic acid in tautomeric form; (**e**) The 3D illustration of (+)-usnic acid bound to Mpro showing interacting amino acids and hydrogen bonds.

The MD simulation of Mpro complexed with (+)-usnic acid was monitored and is shown in Fig. 3. Protein and ligand RMSD are shown in Fig. 3a,b. The RMSD of the alpha-carbon (C_α_) of the Mpro protein reached up to 3.5 Å in 30 ns and then remained equilibrated for the rest of measurement times. The RMSD fluctuation of C_α_ remained within 1–3 Å, which is acceptable for small and globular proteins. The ligand RMSD rapidly rose to 5 Å in 20 ns. Then, the fluctuation of ligand RMSD remained in a steady-state between 4.8 and 5.6 Å for the other monitoring period.

Through the MD simulation, the amino acid of Mpro protein, that (+)-usnic acid mainly interacts with, was profiled and shown in Fig. 3c-d. The interaction through the H-bond and water bridges was predominant and there were slight hydrophobic interactions as well; however, ionic interaction was not detected. His164, Asp187 and Gln189 were the amino acids with the interaction fractions over 0.8, in which most of them were accounted by H-bonds and water bridges. According to the percentage interaction illustration, (+)-usnic acid interacted with Asp187 at most with 78%, followed by 65% of His164. At the 3D model in Fig. 3e, (+)-usnic acid bound to the Mpro binding pocket and interacted with His41, Cys145, Asp187 and Gln189 through hydrogen bonds.

### Anti-SARS-CoV-2 efficacy of (+)-usnic acid and its salts

The anti-SARS-CoV-2 efficacy of (+)-usnic acid salts, sodium usnate (NaU) and potassium usnate (KU), was evaluated. NaU showed a slightly improved efficacy compared to those of the (+)-usnic acid and KU. The IC_50_ of NaU and KU was 5.33 and 7.57 μM respectively, which were lower than that of (+)-usnic acid. The CC_50_ of all compounds remained over 50 μM. The SI of (+)-usnic acid salts was 9.38 for NaU and 6.60 for KU, both showing slightly improved values than (+)-usnic acid (Table 4).

**Table 4:** Anti-SARS-CoV-2 efficacy of (+)-usnic acid and its salts (NaU and KU).

Compound	IC_50_ (μM)	CC_50_ (μM)	SI
(+)-Usnic acid	7.99 (SE ± 0.23)	> 50	6.26
NaU	5.33 (SE ± 0.01)	> 50	9.38
KU	7.57 (SE ± 1.27)	> 50	6.60

### Inhibition of the SARS-CoV-2 variants by (+)-usnic acid

The antiviral efficacy of (+)-usnic acid against SARS-CoV-2 differs depending on the variants. The IC_50_ of (+)-usnic acid against alpha and delta variants were 6.05 μM and 7.17 μM respectively, which were similar to the efficacy against the original strain. However, usnic acid showed enhanced efficacy against the beta variant where the IC_50_ was 2.92 μM. The SI for the beta variant was 11.1, indicating more than twice the SI of the other two variants assayed (Table 5).

**Table 5:** The antiviral efficacy of (+)-usnic acid and controls against the SARS-CoV-2 variants [Alpha (B.1.1.7), Beta (B.1.351), and Delta (B.1.617.2)].

Compounds	(+)-Usnic Acid			Chloroquine		Remdesivir		Lopinavir	
Variants	IC_50_ (μM)	CC_50_ (μM)	SI	IC_50_ (μM)	CC_50_ (μM)	IC_50_ (μM)	CC_50_ (μM)	IC_50_ (μM)	CC_50_ (μM)
SARS-CoV-2 Alpha var (B.1.1.7)	6.05 (SE ± 0.33)	34.8 (SE ± 1.9)	5.8	6.16 (SE ± 0.30)	> 150	2.25 (SE ± 0.16)	> 50	10.8 (SE ± 0.9)	> 50
SARS-CoV-2 Beta var (B.1.351)	2.92 (SE ± 0.06)	32.4 (SE ± 1.0)	11.1	2.64 (SE ± 0.49)	> 150	1.47 (SE ± 0.16)	> 50	11.8 (SE ± 0.5)	> 50
SARS-CoV-2 Delta var (B.1.617.2)	7.17 (SE ± 0.59)	> 50	6.97	6.22 (SE ± 0.34)	> 150	6.48 (SE ± 0.40)	> 50	15.3 (SE ± 5.6)	> 50

## Discussion

From a marine natural products in-house database containing 24,669 natural products, (+)-usnic acid, a substance effective against SARS-CoV-2, was discovered as a hit compound through virtual screening and in vitro assays. The thermodynamic properties and amino acid interactions of (+)-usnic acid were analyzed using MD simulation. Furthermore, the efficacy of (+)-usnic acid and its salts against the SARS-CoV-2 variants was evaluated in this study.

The HTVS of natural product libraries allowed the profiling of docking energies for eight SARS-CoV-2 target proteins within a few days. The docking energy was the priority in the selection and compounds exceeding the permitted range of calculated PK properties were removed. Twenty-seven natural products were identified as potential hits, and considering the ease of securing the compounds, four natural products were selected for in vitro evaluation at a single dose. The (+)-usnic acid showed potent efficacy against SARS-CoV-2 and we conducted further experiments with this compound.

Usnic acid (UA) was first reported in 1844, since then many bioactivities have been studied for this dibenzofuran compound and its derivatives^54^. Usnic acids are found mostly in lichens such as *Alectoria*, *Cladonia*, *Evernia*, *Lecanora*, *Ramalina* and *Usnea*^55^. The usnic acid shows wide-ranging bioactivities including antimicrobial, antifungal, antiprotozoal, insecticidal, anti-inflammatory, phytotoxic, anti-inflammatory, and antimitotic activities^56^. Moreover, the usnic acid shows weak antiviral activity against Herpes simplex type1, Polio type1, papiloma and influenza virus A (H1N1)^56,57^. Recently, usnic acid was produced from the marine fungus *Mycosphaerella* sp., which was isolated from marine sediment in Korea with several compounds in similar scaffolds^58^.

The in vitro immunofluorescence test against the SARS-CoV-2 showed that (+)-usnic acid showed efficacy with IC_50_, CC_50_ and SI corresponding to 7.99 μM, more than 50 μM and 6.26 respectively. Compared to the three compounds (remdesivir, lopinavir and chloroquine) used as positive controls, (+)-usnic acid showed a similar profile to remdesivir, which was approved as a treatment for COVID-19 only for an emergency use. (+)-Usnic acid showed lower IC_50_ values than those of lopinavir and chloroquine.

Although the antiviral efficacy of (+)-usnic acid was validated, we did not know the exact protein target. Therefore, we attempted to identify it using MM-GBSA. MM-GBSA was applied to the energy-minimized pose of docked (+)-usnic acid with eight SARS-CoV-2 protein targets. The binding free energy of Mpro protein was the lowest at –52.05 kcal/mol and the energy difference among those of other proteins was at least 10 kcal/mol. Thus, we determined that Mpro was the most plausible SARS-CoV-2 protein target for (+)-usnic acid. For further analysis, the MD simulations of Mpro with (+)-usnic acid were performed for 100 ns. The protein RMSD increased up to 3.5 Å for 30 ns and ligand RMSD rose to 5 Å for same period as well. The ligand moved slightly to inner pocket of Mpro compared to the binding pose of docking analysis. Instead of a hydrogen bond with Gly143, (+)-usnic acid became more stabilized by H-Bonds with Asp187 and Gln189. Afterwards, (+)-usnic acid was remained stabilized for other 100 ns monitoring period. As a result, the most likely target protein of SARS-CoV-2 for (+)-usnic acid is Mpro.

Subsequently, the antiviral efficacy of (+)-usnic acid under various conditions was evaluated. First, the sodium and potassium salts of (+)-usnic acid were prepared. The IC_50_ and SI of NaU were slightly improved, while those of KU remained almost the same as that of (+)-usnic acid. This improvement in the salts could be explained by the enhanced water solubility. The CC_50_ of both salts was greater than 50 μM. Next, the antiviral efficacy of (+)-usnic acid against the SARS-CoV-2 variants, which are causing serious pandemic worldwide, was evaluated. The (+)-usnic acid against the original SARS-CoV-2 strain showed profiles similar to those of the alpha and delta variants, while it exhibited a superior effect on the beta variant. The reason for this is still unclear and further research is needed to understand why (+)-usnic acid exhibits better efficacy against the beta variant, but it is hypothesized that the K90R mutation on Mpro, 95% prevalent in beta affected the protein stability of the Mpro^59,60^. As lys90 is located on the opposite side of the binding site, it does not significantly affect the binding of (+)-usnic acid. But as a result, protein folding of Mpro becomes destabilized and the overall efficacy of (+)-usnic acid against beta variant seem enhanced.

Usnic acid has been widely used as an ingredient for daily necessities including toothpastes and mouthwashes^55^. It has also been used as a dietary supplement for weight loss^61^. Unfortunately, usnic acid exhibits hepatotoxicity because of the uncoupling of oxidative phosphorylation, resulting in the destruction of mitochondrial respiration at very high concentrations^62^. Nevertheless, usnic acid is expected to have sufficient efficacy and safety for the treatment of COVID-19. The maximum period required for COVID-19 recovery is usually 7 days and paxlovid and molnupiravir, approved for emergency use, are employed for five days only. The use of usnic acid within five days may not show any significant toxicity since there are records of human consumption for a relatively long period of over two weeks^63^. Therefore, it is suggested that usnic acid could be used in emergency situations for the treatment of COVID-19, if its in vivo efficacy is proven. Additionally, oral administration is expected to be possible based on a study showing the improved bioavailability of KU in a mouse tumor xenograft model^64^.

In conclusion, this study identified (+)-usnic acid as a potential drug lead against COVID-19 by the HTVS of a marine natural products database. Further, the in vitro efficacy of (+)-usnic acid has been validated in several SARS-CoV-2 variants. The protein target of (+)-usnic acid was explored using MM-GBSA and MD approaches. SARS-CoV-2 Mpro was identified as a potent protein target of (+)-usnic acid. Although there is a toxicity issue on usnic acid, it is easy to be obtained and has a record of its human use. Therefore, we propose (+)-usnic acid as a potential therapeutic tool for COVID-19 treatment, considering the desperate need to end the deadly pandemic.

## Supplementary Information

Supplementary Information.

## Data Availability

The datasets used and/or analyzed during the current study available from the corresponding author on reasonable request.
