# Supplementary material for: (+)-Usnic acid and its salts, inhibitors of SARS‐CoV‐2, identified by using in silico methods and in vitro assay
Source: Sci Rep. 2022 Jul 30;12:13118. doi: 10.1038/s41598-022-17506-3 (PMC9338942; doi:10.1038/s41598-022-17506-3)

(+)-Usnic acid and its salts, inhibitors of SARS‐CoV‐2, identified by using *in silico* methods and *in vitro* assay

**SUPPLEMENTARY INFORMATION**

**Table S1:** the potential hit compounds identified by the HTVS

| **No** | **Compound Name** | **Formula** | **MW** | **Probable target** | **Binding energy** | **Avail-ability** |
| --- | --- | --- | --- | --- | --- | --- |
| 1 | 2-(1H-Pyrrol-2-yl)-4(1H)-quinolinone (Penicinoline E) | **C_13_H_10_N_2_O** | 210.2 | TMPRSS2 | -7.227 |  |
| 2 | 2-Hydroxy-1-[1-[2-(4-hydroxyphenyl)ethyl]-1H-pyrrol-2-yl]ethanone (Alhagifoline A) | **C_14_H_15_NO_3_** | 245.3 | AAK1 | -7.293 |  |
| 3 | (+)-6-(1-Hydroxy-2-phenylethyl)-4-methoxy-2H-pyran-2-one (Aspergillusol) | **C_14_H_14_O_4_** | 246.3 | Mpro | -7.665 |  |
| 4 | (1-Methyl-1H-imidazol-5-yl)-9H-pyrido[3,4-b]indol-1-ylmethanone (Xestomanzamine A) | **C_16_H_12_N_4_O** | 276.3 | Mpro | -8.258 |  |
| 5 | (4,9-Dihydro-3H-pyrido[3,4-b]indol-1-yl)(1-methyl-1H-imidazol-5-yl)methanone (Xestomanzamine B) | **C_16_H_14_N_4_O** | 278.3 | AAK1 | -7.621 |  |
| 6 | 3-[1-[[2-(4-Hydroxyphenyl)ethyl]amino]ethylidene]-6-methyl-2H-pyran-2,4(3H)-dione (Fusamine) | **C_16_H_17_NO_4_** | 287.3 | Mpro | -7.408 |  |
| 7 | (4-Hydroxyphenyl)-9H-pyrido[3,4-b]indol-1-ylmethanone (Eudistomin Y1) | C_18_H_12_N_2_O_2_ | 288.3 | AAK1 | -7.612 |  |
| 8 | 2,3,5,7-Tetrahydro-9-(4-hydroxyphenyl)-6H-dipyrrolo[4,3,2-de:2′,3′-h]quinolin-6-one (Tsitsikammamine A) | **C_18_H_13_N_3_O_2_** | 303.3 | Mpro | -7.379 |  |
| 9 | N-[2-(4-Hydroxyphenyl)ethyl]-α-oxo-1H-indole-3-acetamide (Polyandrocarpamide C) | **C_18_H_16_N_2_O_3_** | 308.3 | Mpro | -7.718 |  |
| 10 | N-[(2E)-3-(2-Amino-1H-imidazol-5-yl)-2-propen-1-yl]-4-bromo-1H-pyrrole-2-carboxamide (Hymenidin) | C_11_H_12_BrN_5_O | 310.2 | Furin | -7.069 |  |
| 11 | 6,7,12,13-Tetrahydro-5H-indolo[2,3-a]pyrrolo[3,4-c]carbazol-5-one (K 252c) | C_20_H_13_N_3_O | 311.3 | TMPRSS2 | -7.401 |  |
| 12 | (6S)-5,6-Dihydro-3,6-di-1H-indol-3-yl-2(1H)-pyrazinone | **C_20_H_16_N_4_O** | 328.4 | Mpro | -8.158 |  |
| 13 | 6-Bromo-1-(3,4-dihydro-2H-pyrrol-5-yl)-9H-pyrido[3,4-b]indol-8-ol (Eudistomidin A) | C_15_H_12_BrN_3_O | 330.2 | AAK1 | -8.578 |  |
| 14 | 7-Bromo-1-(3,4-dihydro-2H-pyrrol-5-yl)-9H-pyrido[3,4-b]indol-6-ol (Eudistomin P) | **C_15_H_12_BrN_3_O** | 330.2 | AAK1 | -8.978 |  |
| 15 | (9bR)-2,6-Diacetyl-7,9-dihydroxy-8,9b-dimethyl-1,3(2H,9bH)-dibenzofurandione ((+)-Usnic acid) | **C_18_H_16_O_7_** | 344.3 | Mpro | -7.70 | ○ |
| 16 | N-[3-(7-Bromo-9H-pyrido[3,4-b]indol-1-yl)propyl]guanidine (Opacaline C) | C_15_H_16_BrN_5_ | 346.2 | AAK1 | -7.527 |  |
| 17 | (4aS,9bR)-6-acetyl-7,9-dihydroxy-3,4a-dimethoxy-8,9b-dimethyl-4H-dibenzofuran-1-one (Mycousfuran A) | C_18_H_20_O_7_ | 348.3 | RdRP | -7.569 | ○ |
| 18 | N-[4-(7-Bromo-9H-pyrido[3,4-b]indol-1-yl)butyl]guanidine (Opacaline A) | C_16_H_18_BrN_5_ | 360.3 | AAK1 | -7.213 |  |
| 19 | 4-(3-Aminopropoxy)-3,5-dibromo-N-methylbenzeneethanamine | **C_12_H_18_Br_2_N_2_O** | 366.1 | Mpro | -7.098 |  |
| 20 | [2-(Dimethylamino)-3,6-dihydroimidazo[4′,5′:4,5]pyrido[2,3-b]indol-4-yl](4-hydroxyphenyl)methanone (Grossularine II) | **C_21_H_17_N_5_O_2_** | 371.4 | RdRP | -7.841 |  |
| 21 | (5bS)-6b,6,7,8-Tetrahydro-2,12-dimethoxy-10H,16H-pyrrolo[2,1-c]quinazolino[3,2-a][1,4]benzodiazepine-10,16-dione (Circumdatin J) | C_21_H_19_N_3_O_4_ | 377.4 | Mpro | -7.739 |  |
| 22 | N-[(2E)-3-(2-Amino-1H-imidazol-4-yl)-2-propenyl]-4,5-dibromo-1H-pyrrole-2-carboxamide (Oroidin) | **C_11_H_11_Br_2_N_5_O** | 389.1 | Helicase | -7.069 |  |
| 23 | Methyl 3-hydroxy-2-[[2-[(3-pyridinylcarbonyl)amino]benzoyl]amino]benzoate (Terremide A) | C_21_H_17_N_3_O_5_ | 391.4 | RdRP | -7.024 |  |
| 24 | 5-(3-Chloro-4-hydroxyphenyl)-2-(3-chloro-4-methoxyphenyl)-3,4,5-trihydroxy-2-cyclopenten-1-one (Sydowin B) | **C_18_H_14_Cl_2_O_6_** | 397.2 | RdRP | -7.808 |  |
| 25 | N-[(2E)-3-(2-Amino-1H-imidazol-5-yl)-2-propen-1-yl]-4,5-dibromo-1-methyl-1H-pyrrole-2-carboxamide (Sventrin) | **C_12_H_13_Br_2_N_5_O** | 403.1 | TMPRSS2 | -7.347 |  |
| 26 | 3-[[3-[(1S,3S,4S,5aS,9S,9aR)-1,4,5,8,9,9a-Hexahydro-3,9-dimethyl-8-oxo-3H-1,4:3,5a-dimethano-2-benzoxepin-9-yl]-1-oxopropyl]amino]-2,4-dihydroxybenzoic acid (Platensimycin) | **C_24_H_27_NO_7_** | 441.5 | RdRP | -7.371 | ○ |
| 27 | 1,11-Dichloro-12,13-dihydro-12-(4-O-methyl-β-D-glucopyranosyl)-5H-indolo[2,3-a]pyrrolo[3,4-c]carbazole-5,7(6H)-dione (Rebeccamycin) | **C_27_H_21_Cl_2_N_3_O_7_** | 570.4 | Mpro | -7.818 | ○ |

**Table S2:** the inhibition of infection percentage of four hit compounds against SARS-CoV-2

|  | **Concentration**  **(μM)** | **Inhibition of infection**  **(%)** | **Cell number to mock**  **(%)** |
| --- | --- | --- | --- |
| (+)-Usnic acid | 10 | 61.63 | 94.69 |
| Mycousfuran A | 10 | 0.96 | 109.23 |
| Platensimycin | 10 | 3.16 | 109.23 |
| Rebeccamycin | 10 | 10.62 | 72.32 |

**Figure S1:** Screening flowchart and criterion used in (+)-usnic acid selection

**
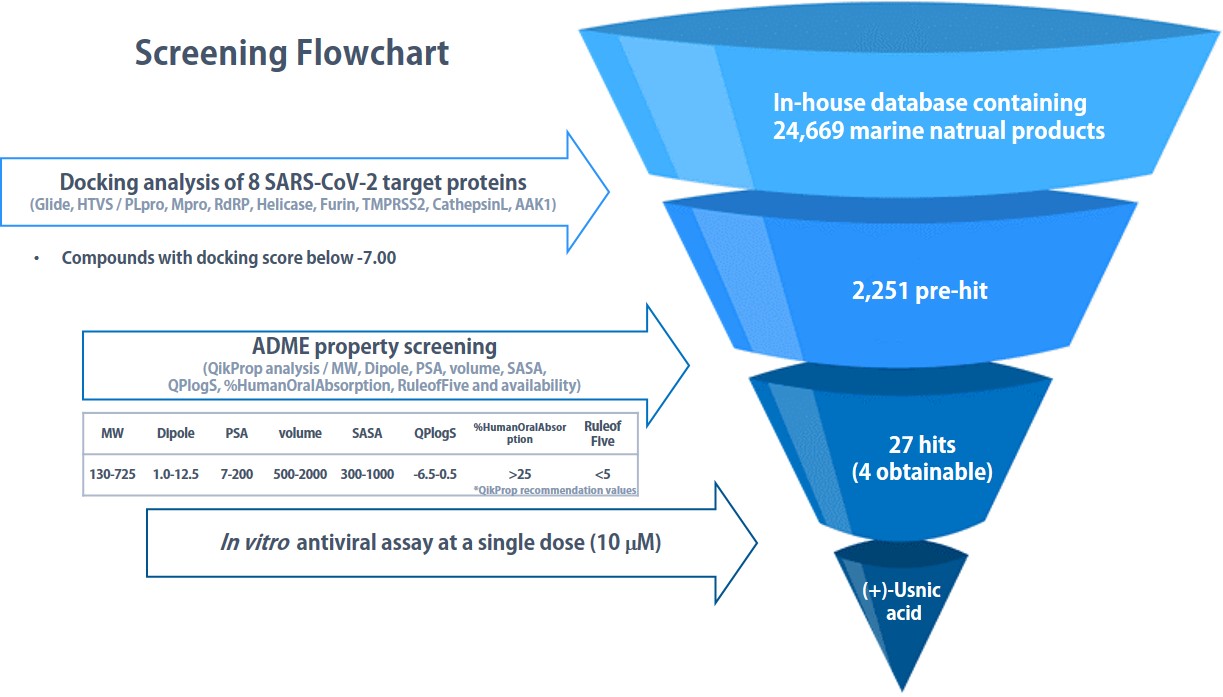
**

**Figure S2:** Possible (+)-usnic acid tautomeric formations

**
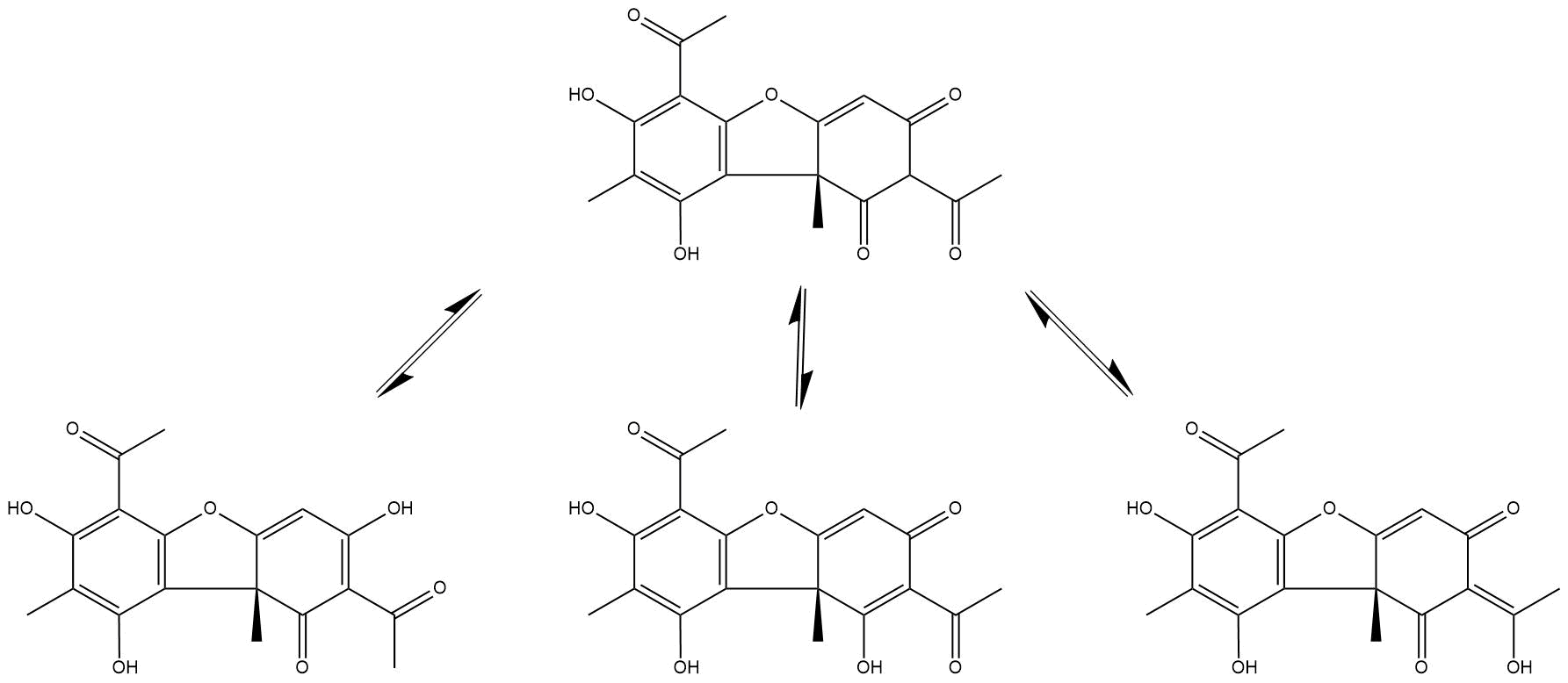
**

**Figure S3:** *In-vitro* antiviral activities of four hit compounds against SARS-CoV-2 at single concentration


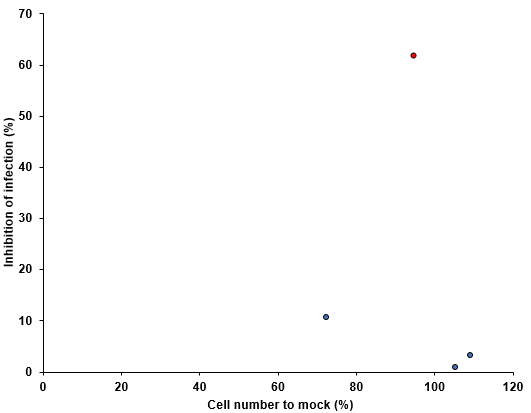


**Figure S4:** Fluorescent images of (+)-usnic acid, usnate salts and control (remdesivir)


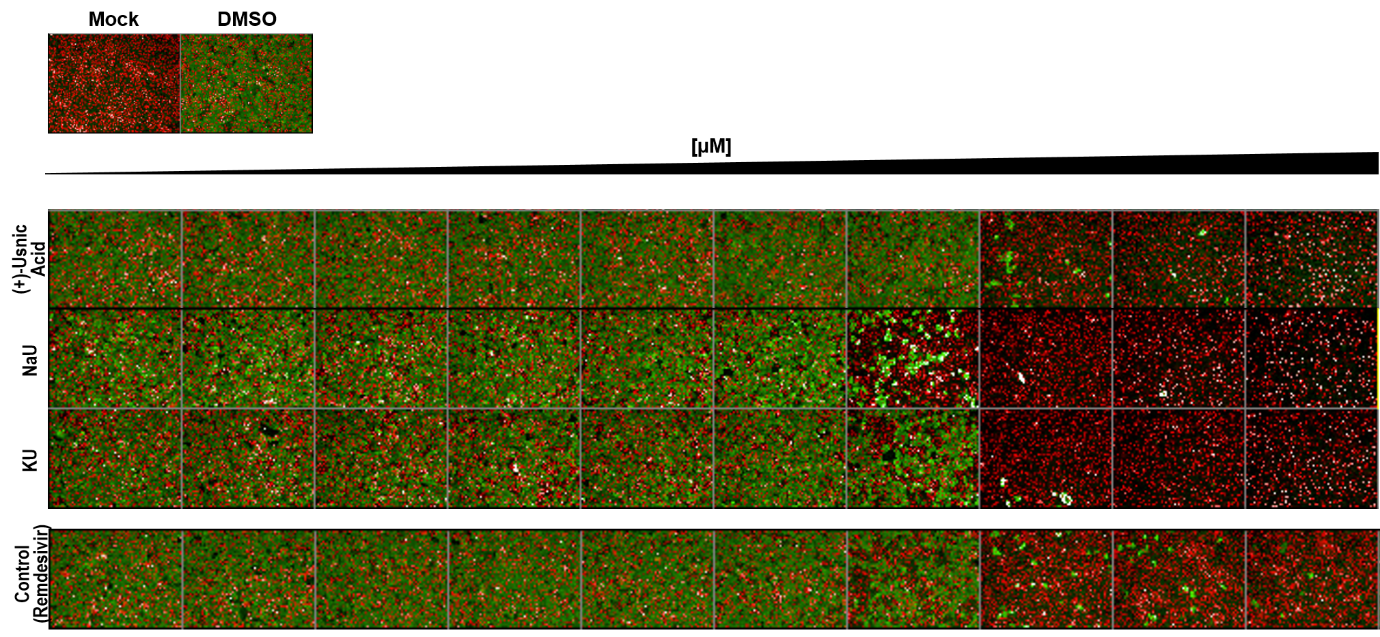


**Figure S5:** The Glide docking 2D interaction illustration of (+)-usnic acid with Mpro


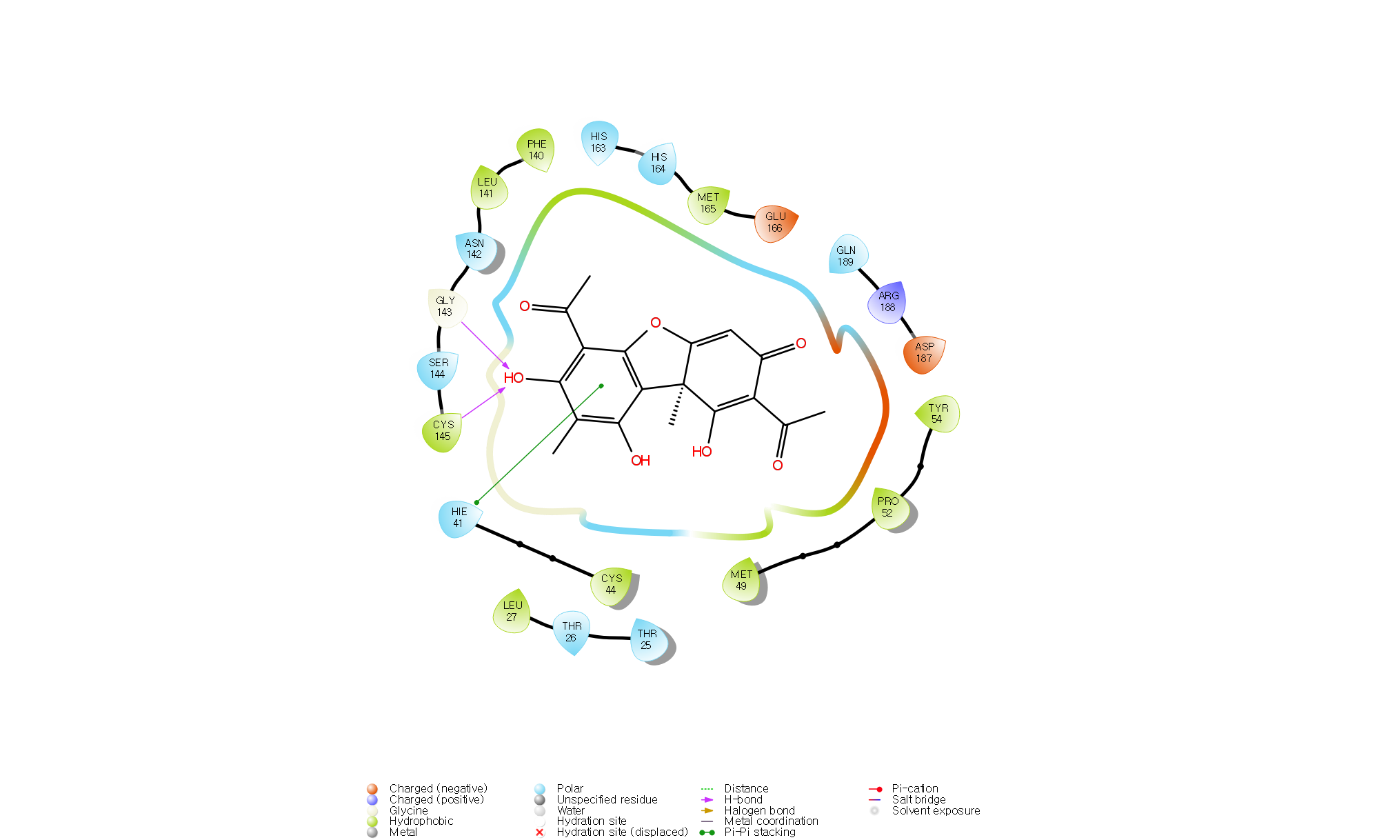


**Figure S6:** The 3D overlay of Glide docking and MD stable trajectory orientation of (+)-usnic acid with Mpro (yellow – docking conformation, green – MD stable conformation)


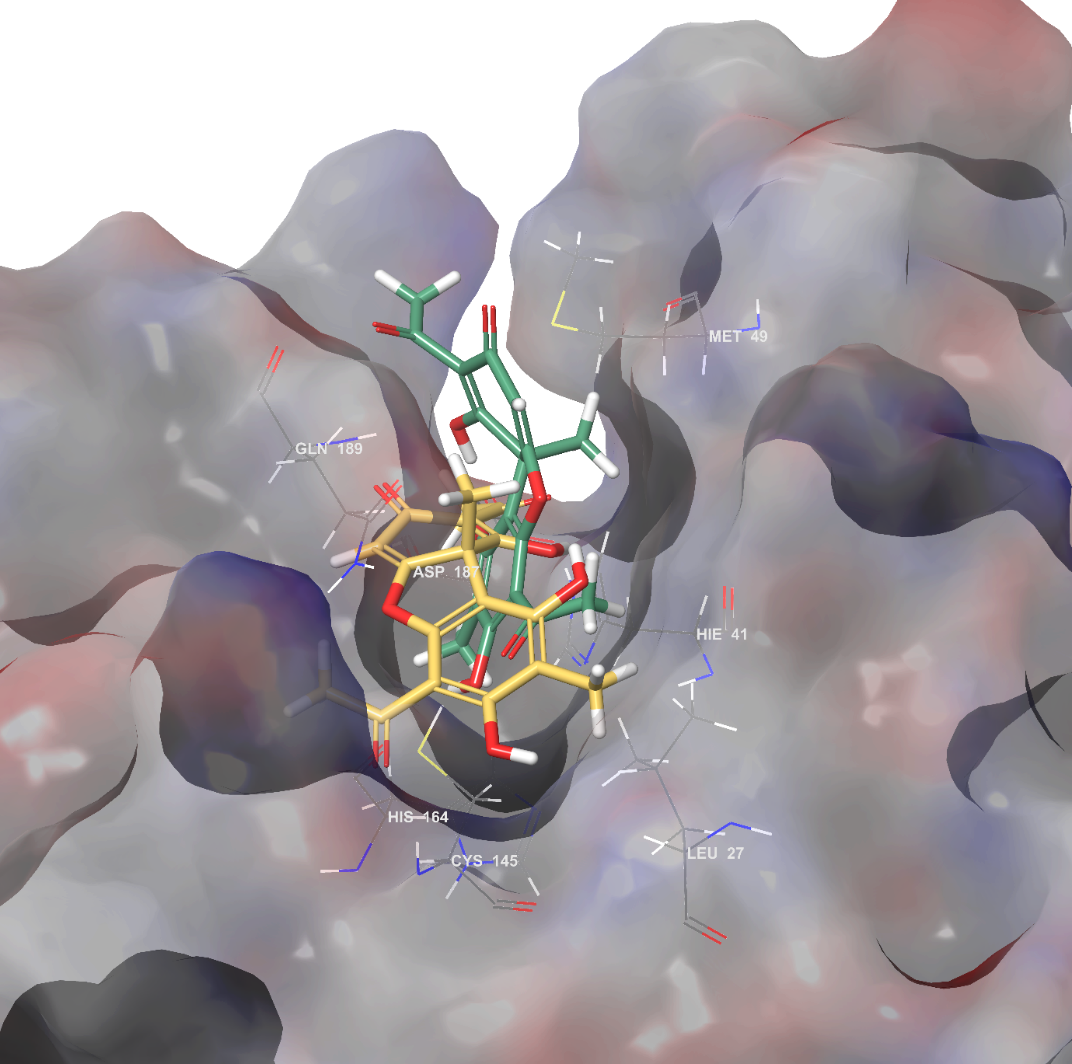


**Figure S7:** ^1^H and ^13^C NMR spectra of (+)-Usnic acid

**(+)-Usnic acid**: [α]^25^_D_ = 500.4 (c 1.0, CHCl_3_, reference [α]^25^_D_ = 488); ^1^H NMR (CDCl_3_, 700 MHz) δ 11.00 (1H, s), 5.96 (1H, s), 2.66 (6H, s), 2.08 (3H, s) 1.74 (3H, s); ^13^C NMR (CDCl_3_, 175 MHz) δ 201.9, 200.5, 198.2, 191.9, 179.5, 164.0, 157.6, 155.4, 109.4, 105.4, 104.1, 101.7, 98.5, 59.2, 32.3, 31.4, 28.1, 7.7; ESIMS m/z 345.1 [M + H]^+^.


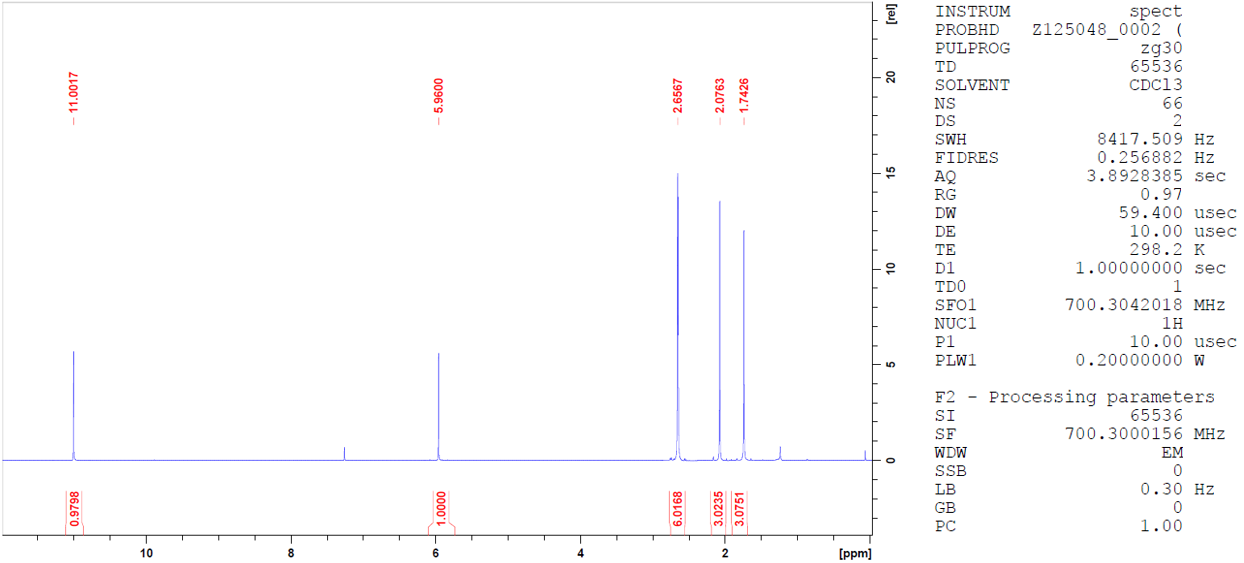

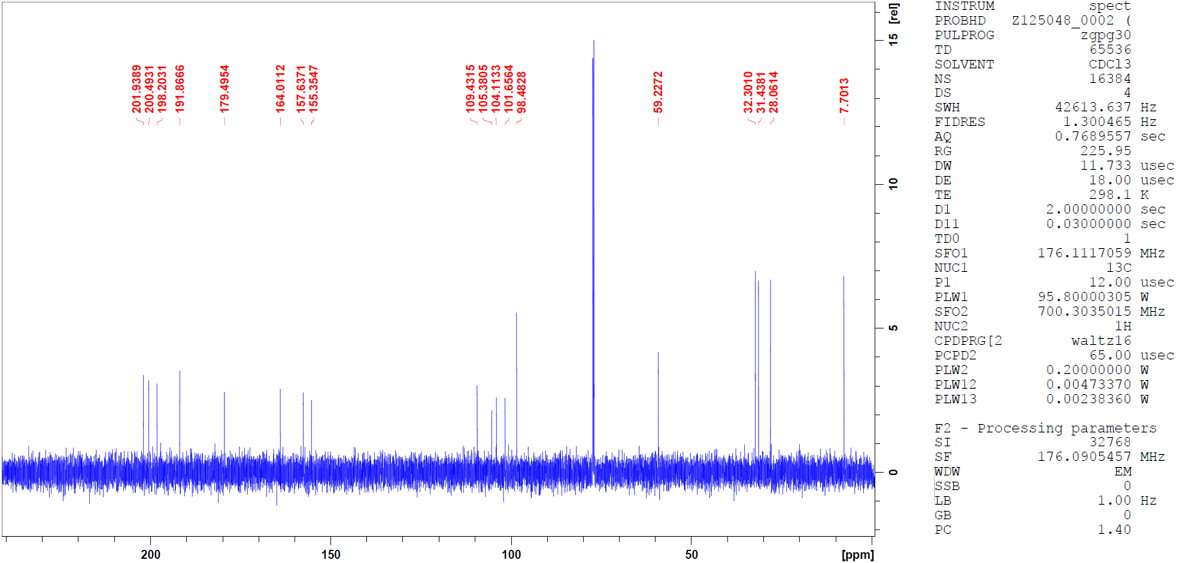


**Figure S8:** ^1^H NMR spectra of sodium and potassium usnate

**Sodium Usnate(NaU)**: ^1^H NMR (DMSO-*d*_6_, 700 MHz) δ 5.57 (1H, s), 2.58 (3H, s), 2.20 (3H, s), 1.90 (3H, s) 1.53 (3H, s).


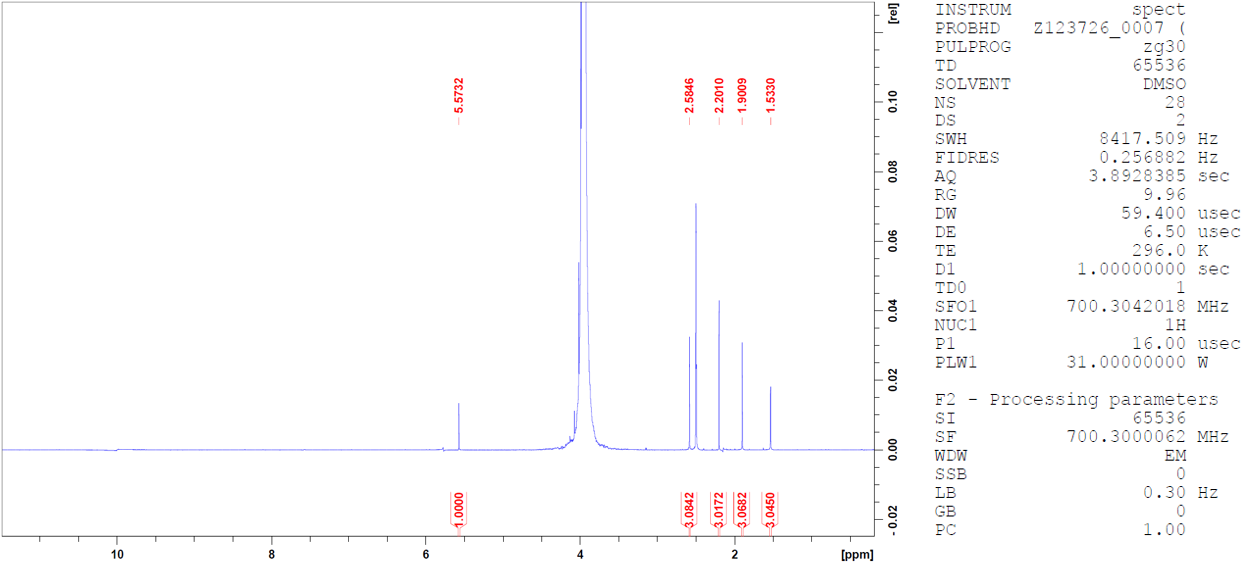


**Potassium Usnate(KU)**: ^1^H NMR (DMSO-*d*_6_, 700 MHz) δ 5.57 (1H, s), 2.58 (3H, s), 2.20 (3H, s), 1.90 (3H, s) 1.53 (3H, s).


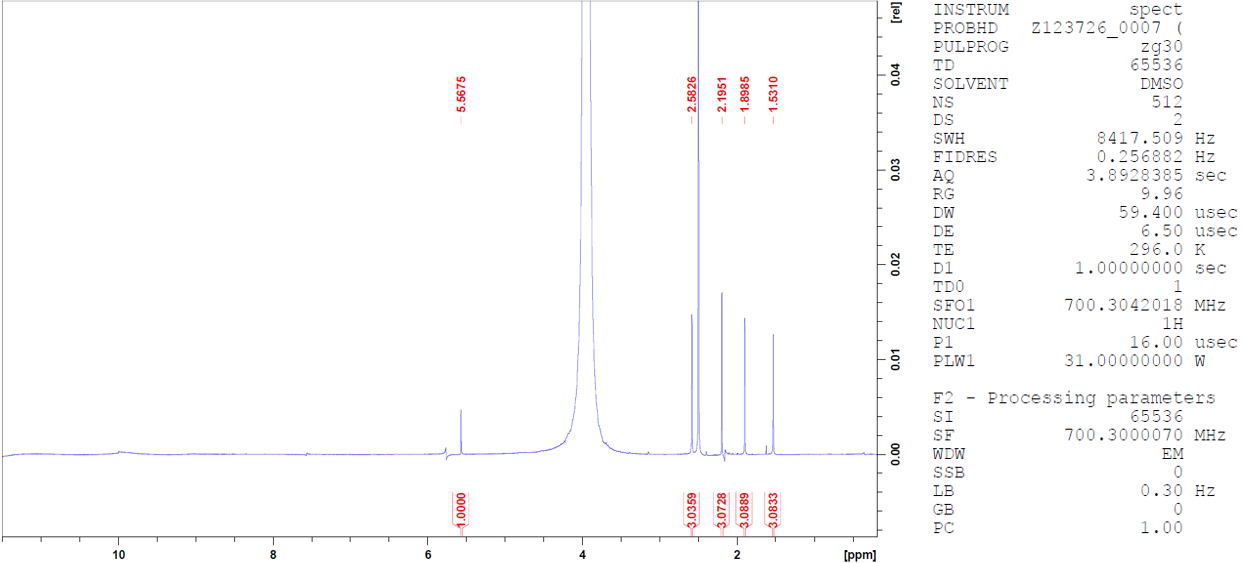


**Figure S9:** Purity of (+)-usnic acid analyzed using HPLC

The purity of the (+)-usnic acid was confirmed using Agilent Technologies 1260 series HPLC. Phenomenex luna C18 (100 mm x 4.6 mm, 5 μm) column with 65% CH_3_CN in H_2_O was used for HPLC. The uv length 280 nm was monitored for 15 min. The purity of (+)-usnic acid was over 99%.


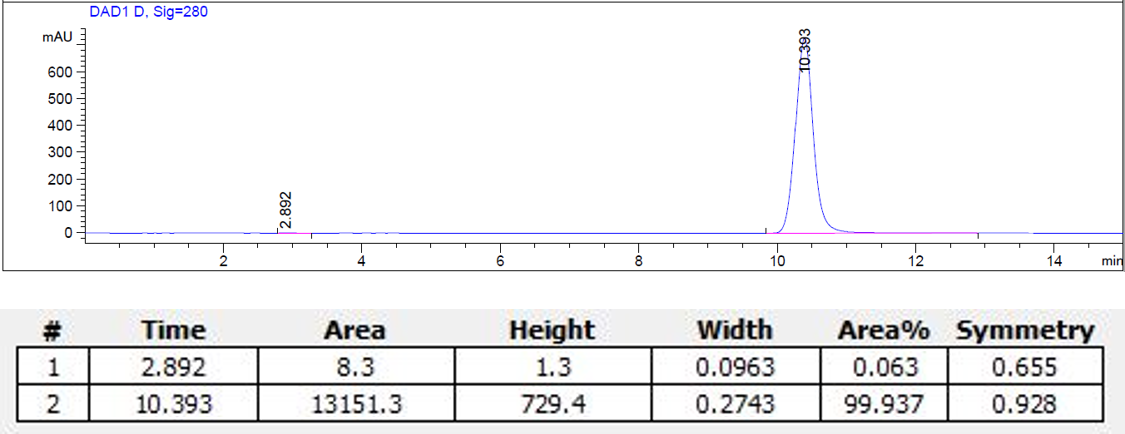

Supplement: Supplementary file 1 — Supplementary Information. [file 41598_2022_17506_MOESM1_ESM.docx]
